# T1AM/TAAR1 System Reduces Inflammatory Response and β-Amyloid Toxicity in Human Microglial HMC3 Cell Line

**DOI:** 10.3390/ijms241411569

**Published:** 2023-07-17

**Authors:** Beatrice Polini, Caterina Ricardi, Andrea Bertolini, Vittoria Carnicelli, Grazia Rutigliano, Federica Saponaro, Riccardo Zucchi, Grazia Chiellini

**Affiliations:** 1 Department of Pathology, University of Pisa, 56100 Pisa, Italy; c.ricardi@student.unisi.it (C.R.); a.bertolini@student.unisi.it (A.B.); vittoria.carnicelli@unipi.it (V.C.); federica.saponaro@unipi.it (F.S.); riccardo.zucchi@unipi.it (R.Z.); 2 Institute of Clinical Sciences, Imperial College London, London SW7 2AZ, UK; grazia.rutigliano.gr@gmail.com

**Keywords:** neurodegeneration, microglia, inflammation, neuroprotection, trace amine-associated receptors type 1 (TAAR1), 3-iodothyronamine

## Abstract

Microglial dysfunction is one of the hallmarks and leading causes of common neurodegenerative diseases (NDDs), including Alzheimer’s disease (AD) and Parkinson’s disease (PD). All these pathologies are characterized by aberrant aggregation of disease-causing proteins in the brain, which can directly activate microglia, trigger microglia-mediated neuroinflammation, and increase oxidative stress. Inhibition of glial activation may represent a therapeutic target to alleviate neurodegeneration. Recently, 3-iodothyronamine (T1AM), an endogenous derivative of thyroid hormone (TH) able to interact directly with a specific GPCR known as trace amine-associated receptor 1 (TAAR1), gained interest for its ability to promote neuroprotection in several models. Nevertheless, T1AM’s effects on microglial disfunction remain still elusive. In the present work we investigated whether T1AM could inhibit the inflammatory response of human HMC3 microglial cells to LPS/TNFα or β-amyloid peptide 25–35 (Aβ25–35) stimuli. The results of ELISA and qPCR assays revealed that T1AM was able to reduce microglia-mediated inflammatory response by inhibiting the release of proinflammatory factors, including IL-6, TNFα, NF-kB, MCP1, and MIP1, while promoting the release of anti-inflammatory mediators, such as IL-10. Notably, T1AM anti-inflammatory action in HMC3 cells turned out to be a TAAR1-mediated response, further increasing the relevance of the T1AM/TAAR1 system in the management of NDDs.

## 1. Introduction

Microglial cells, the brain-resident immune cells, play a pivotal role in regulating the cellular processes involved in the development of neuronal networks and the homeostasis maintenance of the central nervous system (CNS) [1]. Notably, in response to abnormal microenvironment factors, including, for example, stress, aging, injuries, infections, and hypoxia–ischemia, microglia can assume different activation states, from neuroprotective to neurodestructive, the equilibrium of which may contribute to the onset and outcome of neuroinflammatory and neurodegenerative mechanisms [2]. Indeed, dysfunction of microglia is one of the hallmarks and leading causes of common neurodegenerative diseases (NDDs), such as Alzheimer’s disease (AD), Parkinson’s disease (PD), Huntington’s disease (HD), and amyotrophic lateral sclerosis (ALS) [3,4,5,6,7]. All these pathologies are characterized by aberrant aggregation of disease-causing proteins in the brain [8], which can directly activate microglia, trigger microglia-mediated neuroinflammation, and increase oxidative stress [9].

**Figure 1:**
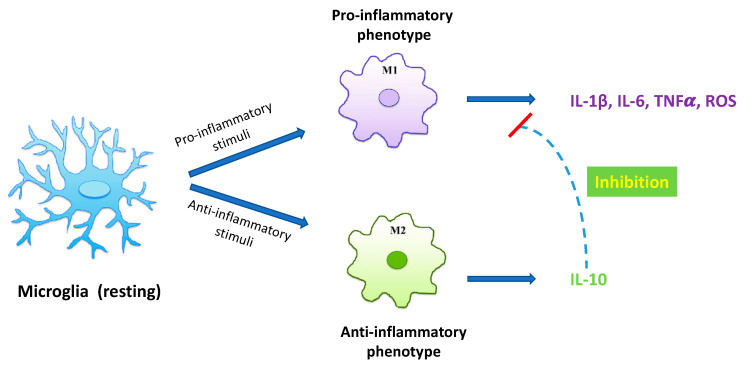
Diagram of microglia activation. Resting microglia may turn into distinct phenotypes, namely, M1 phenotype and M2 phenotype. M1 classical state releases proinflammatory cytokines and cytotoxic substances, including IL-1ß, IL-6, TNFα, and ROS, inducing neurological damage. On the other hand, the M2 alternative state produces anti-inflammatory cytokines, such as IL-10, able to inhibit the production of proinflammatory cytokines by microglia, thus exerting a neuroprotective role in the CNS.

Under physiological conditions microglial cells maintain a ramified cell shape, defined as “resting state” (Figure 1), and vigilantly monitor and protect neuronal function. In the event of brain injury, infection, or neurodegeneration, microglia adopt a phagocytic phenotype, defined as “M1 state”, in which the cell attains an amoeboid morphology, and may secrete a wide variety of neurotoxic factors, including proinflammatory cytokines, proteinases, and reactive oxygen species (ROS), leading to exacerbated damage and neuronal death [10]. Accumulating evidence suggests that the initial classical activation of microglia is followed by a secondary alternative activation, defined as “M2 state”, which is important for neuronal cell repair, tissue remodeling, and suppression of inflammation [11] (Figure 1). Since chronic inflammatory activation of microglia is correlated with common NDDs, functional modulation of microglial phenotypes could represent a valid therapeutic strategy [10,12,13]. 

Recently, 3-iodothyronamine (T1AM), an endogenous thyroid hormone (TH) derivative, able to interact directly with a specific G-protein coupled receptor known as trace amine-associated receptor 1 (TAAR1) [14,15], has gained interest for its ability to promote neuroprotective effects in several models, including seizure-related excitotoxic damage, altered autophagy, amyloidosis, and OGD-induced synaptic dysfunction [16,17,18,19], and to efficiently cross the blood–brain barrier (BBB) [20]. TAAR1 is an established regulatory protein expressed and broadly distributed in the brain monoaminergic systems [21], which include the dopaminergic system, the noradrenergic system, and the serotonergic system [22]. Collectively, all these systems play important roles in mood, cognition, emotion, reward, learning, attention, and motor activity [23], and their dysregulation is associated with a variety of neurological and neurodegenerative disorders, several of which are among the leading causes of death and disability worldwide [24]. Recent studies revealed that pharmacologically targeting TAAR1 within the CNS has resulted in successful clinical trials for the treatment of schizophrenia, depression, addiction, and NDDs [25,26,27,28]. More recently, TAAR1 expression and functionality in immune system regulation and immune cell activation has become a topic of emerging interest [29]; nevertheless, so far, few studies have examined the role of TAAR1 in CNS-resident neuroimmune cells [30,31]. Notably, a recent report highlighted the expression of TAAR1 in macrophages/microglia bordering multiple sclerosis (MS) lesions [32], supporting TAAR1 as a novel pharmacological target in cells directly implicated in neuroinflammation.

On these premises, we directed our attention to examining the potential of the T1AM/TAAR1 signaling system in regulating brain neuroinflammatory responses. Using HMC3 human microglia cells as an in vitro model [33], we demonstrated, for the first time, that T1AM inhibits LPS/TNFα-induced inflammatory response through the activation of TAAR1. Since it is well known that fibrillar amyloid-β (Aβ) peptides play an important role in microglial activation in AD [34], we also showed that the T1AM-TAAR1 signaling pathway was able to protect against Aβ-induced cytotoxic and inflammatory responses in HMC3 cells, and that TAAR1 stimulation can inactivate microglial NF-κB signaling.

Even though still at a preliminary level, the results of our study highlight the ability of T1AM to extensively modulate microglia-mediated neuroinflammation, providing further insight into its possible therapeutic application for the prevention of neurodegeneration.

## 2. Results

### 2.1. T1AM Decreased the Inflammatory Phenotype of LPS/TNFα-Stimulated HMC3 Human Microglial Cells

As previously reported [35,36,37], exposure of human microglial clone 3 (HMC3) cells to LPS/TNFα stimulus for 24 h resulted in a significant increase in proinflammatory IL-6 release in cell culture media, while no effect on anti-inflammatory IL-10 levels was observed as compared with control cells. In the present study, HMC3 microglial cell line was used as an in vitro model to investigate the ability of T1AM to prevent neuroinflammation.

**Figure 2:**
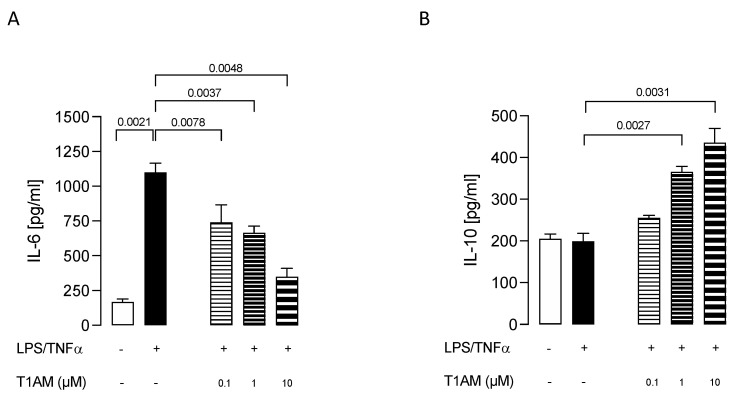
Release of pro- and anti-inflammatory interleukins, IL–6 (**A**) and IL–10 (**B**), induced by different concentrations of T1AM. Data represent means ± S.E.M. from three independent experiments (*n* = 3), performed in duplicate. Statistical analysis was performed by ordinary one-way ANOVA followed by Dunnett’s multiple comparison test. For each experimental condition a selected specific pattern has been used.

**Figure 3:**
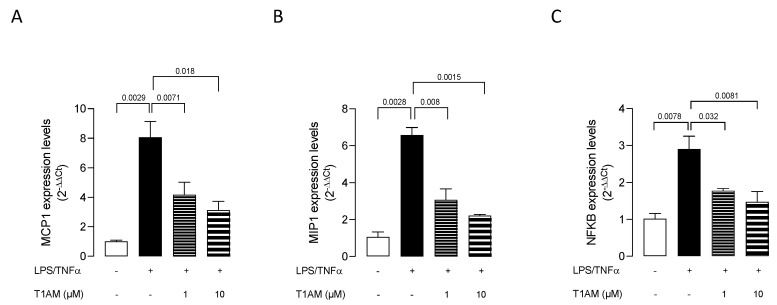
T1AM decreases the expression of inflammatory-response-related genes stimulated by LPS/TNFα treatment in HMC3 cells. (**A**) monocyte chemoattract protein–1 (MCP1), (**B**) macrophage inflammatory protein–1 (MIP1), and (**C**) transcription factor NF-kB. Data represent means ± S.E.M. from three independent experiments (*n* = 3), performed in duplicate. Statistical analysis was performed by ordinary one-way ANOVA followed by Dunnett’s multiple comparison test. For each experimental condition a selected specific pattern has been used.

Dose–response experiments were carried out by exposing HMC3 cells to pretreatment with increasing concentrations (0.1, 1, and 10 μM) of T1AM for 1 h, followed by LPS/TNFα treatment for 24 h. ELISA assays on cell culture media revealed that T1AM causes a significant (*p* < 0.05) dose-dependent reduction of IL-6 levels as compared to LPS/TNFα-treated cells (Figure 2A). A significant (*p* < 0.05) dose-dependent increase in IL-10 levels was also observed in the same set of experiments (Figure 2B), suggesting that T1AM pretreatment may temper hyperinflammation. Moreover, gene expression analysis revealed that pretreatment with 1 or 10 μM T1AM significantly decreases the expression of inflammatory-response-related genes in LPS/TNFα-treated cells, including the monocyte chemoattract protein-1 (MCP1), the macrophage inflammatory protein-1 (MIP1), and the transcription factor NF-kB (Figure 3).

**Figure 4:**
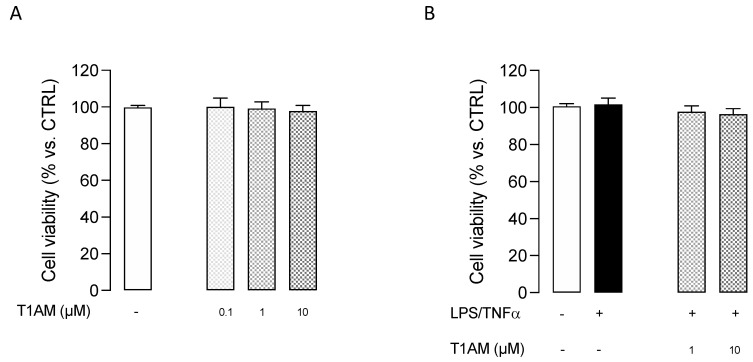
MTT assay performed with different concentrations of T1AM. (**A**) Effect of T1AM on HMC3 Cell viability. (**B**) Effect of T1AM on the viability of LPS/TNFα–treated cells. Data represent means ± S.E.M. from three independent experiments, performed in triplicate (*n* = 3). Statistical analysis was performed by ordinary one-way ANOVA followed by Tukey’s multiple comparison test. For each experimental condition a selected specific pattern has been used.

In addition, T1AM was nontoxic to microglial cells when used at 0.1, 1, and 10 μM concentrations for 24 h (Figure 4).

### 2.2. T1AM Uptake and Metabolism in HMC3 Human Microglial Cells

T1AM is known to be transported inside cells and rapidly transformed into 3-iodothyroacetic acid (TA1) [20,38,39].

Liquid chromatography with tandem mass spectrometry (LC–MS/MS) was used to measure T1AM and TA1 levels in HMC3 cells. After incubating HMC3 cell preparations with 0.1 µM T1AM for 5, 15, 30, and 60 min at 37 °C, the cell culture media were collected, and cell lysates were prepared according to a previously reported procedure [40]. 

As shown in Table 1, T1AM was rapidly taken up by HMC3 cells and catabolized to TA1, indicating that in HMC3 cells, amine oxidases were metabolically active. Notably, the product of T1AM catabolism, TA1, after forming was released from cells, and detected in the collected cell culture media, showing increasing concentrations over time. In HMC3 cell lysates, the concentration of T1AM appeared to remain constant over time at values corresponding to approximately 25% of the administered dose, whereas only a negligible amount of TA1 was found.

### 2.3. Trace Amine-Associated Receptor TAAR1 Is Involved in T1AM-Mediated Anti-Inflammatory Response of Microglial Cells

The evidence that T1AM was able to suppress the response of microglia cells to inflammatory stress prompted us to extend our investigation to its mechanism of action. 

It is known from the literature that T1AM is a high-affinity ligand for TAAR1 [14], and TAAR1 was demonstrated to mediate T1AM’s protective effect against synaptic plasticity abnormalities in a mouse model of AD [41]. Therefore, we explored whether TAAR1 could also be involved in T1AM-mediated anti-inflammatory response of HMC3 cells. 

**Figure 5:**
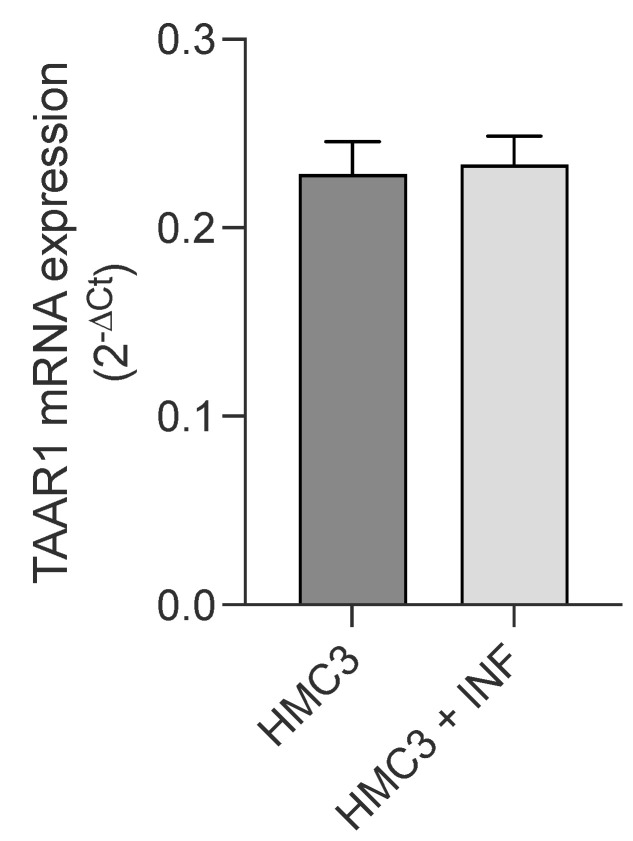
Real-time quantification of TAAR1 expression in HMC3 cells before and after exposure to LPS (10 μg/mL)/TNFα (50 ng/mL) treatment for 24 h. Data represent means ± S.E.M. from three independent experiments, performed in triplicate (*n* = 3). Statistical analysis was performed by ordinary Student’s *t*-test.

First, expression of TAAR1 in HMC3 microglial cells was assessed by qPCR analysis. As shown in Figure 5, resting microglial cells showed TAAR1 expression, and no changes were observed after 24 h treatment with LPS/TNFα.

Then, we investigated whether TAAR1 could play a role in our model of inflamed microglia by using a TAAR1 selective antagonist (EPPTB) and a selective agonist (RO5166017) [42].

**Figure 6:**
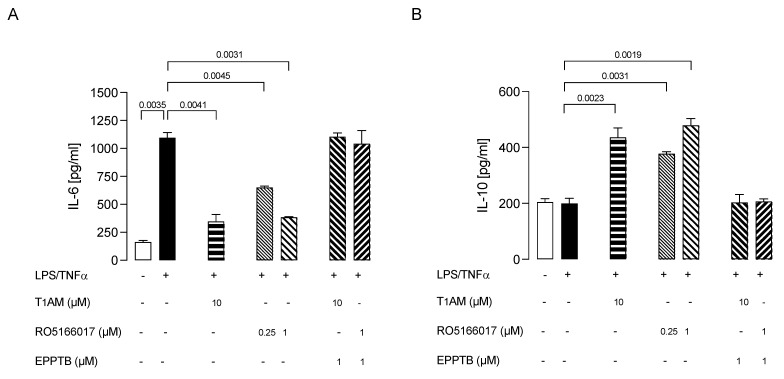
Ability of T1AM to decrease the inflammatory phenotype of LPS/TNFα-stimulated HMC3 cells by the modulation of TAAR1. Bars represent the release (pg/mL) of IL–6 (**A**) and IL–10 (**B**) in the presence of the drugs at the indicated concentrations. Data represent means ± S.E.M. from independent experiments (*n* = 3), performed in duplicate. Statistical analysis was performed by ordinary one-way ANOVA followed by Dunnett’s multiple comparison test. For each experimental condition a selected specific pattern has been used.

We observed that T1AM’s protective effect against microglia activation was abolished by coadministration of TAAR1 selective antagonist EPPTB (5 nM) (Figure 6A,B). Conversely, the administration of the TAAR1 agonist RO5166017 (1 μM) to LPS/TNFα-stimulated HMC3 cells mimicked the effects on the release of both IL-6 and IL-10 previously observed after administering T1AM (10 μM) (Figure 6A,B). 

### 2.4. 3-Iodothyroacetic Acid (TA1) Was Not Able to Decrease the Inflammatory Phenotype of LPS/TNFα-Stimulated HMC3 Human Microglial Cells 

**Figure 7:**
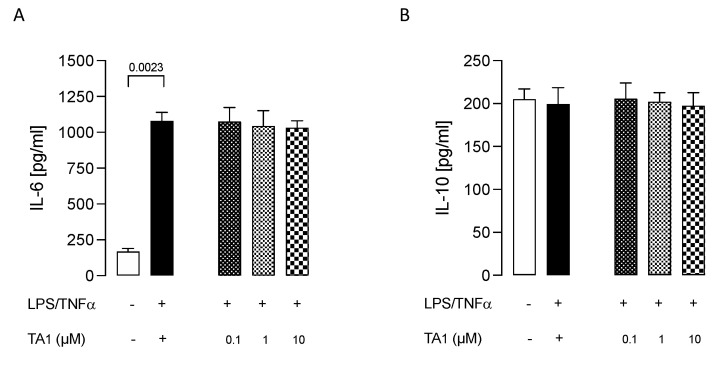
3-iodothyroacetic acid TA1 does not decrease the inflammatory phenotype of LPS/TNFα–stimulated HMC3 cells. After pretreatment with TA1 at the indicated concentrations the release of IL–6 (**A**) and IL–10 (**B**) from LPS/TNFα–stimulated HMC3 cells were examined. Data represent means ± S.E.M. from three independent experiments (*n* = 3), performed in duplicate. Statistical analysis was performed by ordinary one-way ANOVA followed by Dunnett’s multiple comparison test. For each experimental condition a selected specific pattern has been used.

Since 3-iodothyroacetic acid (TA1), the major catabolite of T1AM [43,44,45], has been reported to be responsible for some effects elicited after the administration of exogenous T1AM, we checked whether TA1 administration could also decrease the inflammatory phenotype of LPS/TNFα-stimulated HMC3 cells. We observed that administration of TA1 (0.1, 1, and 10 μM) to LPS/TNFα-stimulated HMC3 cells was not able to produce any significant effect on both IL-6 and IL-10 release from cells (Figure 7), suggesting that the decreased activation of microglia is exclusively due to the action of T1AM through the interaction with the TAAR1 receptor.

### 2.5. T1AM-TAAR1 System Protects against Aβ_25–35_-Mediated Release of Proinflammatory Factors in HMC3 Cells

Studies have revealed that Aβ oligomers activate microglia to secrete proinflammatory factors, including cytokines, chemokines, complements factors, and a large variety of free radicals [46,47]. Suppressing the response of microglia cells to inflammatory stress may attenuate AD pathology and lessen the disease progression [48,49].

**Figure 8:**
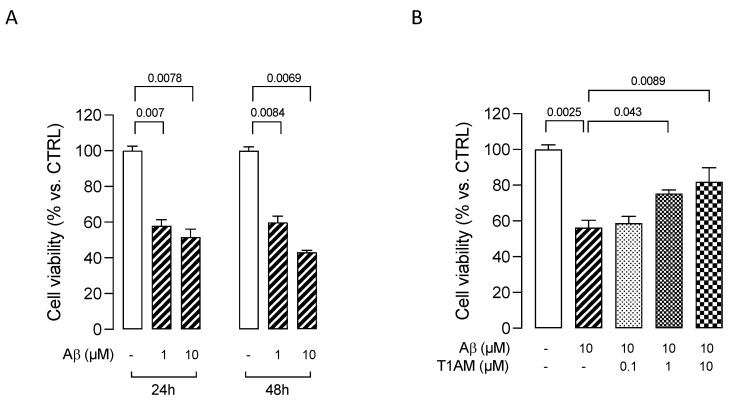
MTT assays in HMC3 cells exposed to Aβ. (**A**) Effects on HMC3 cells’ viability of treatment with β–amyloid peptide 25–35 (Aβ) at 1 and 10 μM for 24 or 48 h. (**B**) Effects on HMC3 cells’ viability of pretreatment with T1AM at 0.1, 1, and 10 μM, followed by exposure to 10µM Aβ for 24 h. Data represent means ± S.E.M. from three independent experiments, performed in triplicate (*n* = 3). Statistical analysis was performed by ordinary one-way ANOVA followed by Dunnett’s multiple comparison test. For each experimental condition a selected specific pattern has been used.

We initially assessed the effects of β-amyloid peptide 25–35 (Aβ25–35) [50] on HMC3 cells’ viability. The cells were treated with two different concentrations (1 and 10 μM) of Aβ25–35 for 24 or 48 h. Aβ25–35 was found to induce cytotoxicity in HMC3 cells in a concentration- and time-dependent manner, with a 10 μM concentration promoting a 50% reduction in cell viability after 24 h (Figure 8A). We thus used this condition to evaluate the modulation of Aβ25–35 cytotoxicity in further experiments. HMC3 cells were treated with 10 μM Aβ25–35 in the presence of different concentrations (0.1, 1, and 10 μM) of T1AM for 24 h to determine the effects of T1AM on β-amyloid-induced cytotoxicity. In line with expectations, MTT assays indicated that T1AM was able to protect cells from Aβ-induced cytotoxicity in a concentration-dependent manner, with the identified best concentrations of 1 and 10 μM being used in further experiments (Figure 8B).

**Figure 9:**
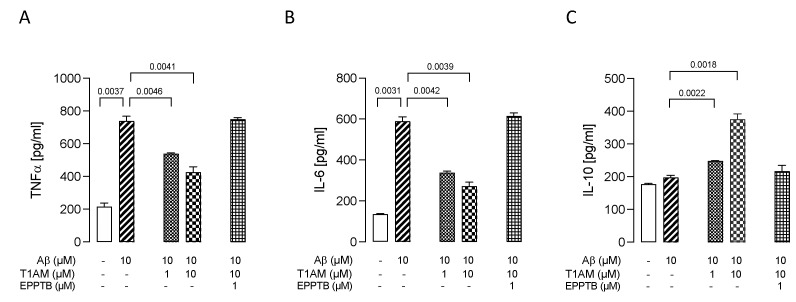
T1AM pretreatment modulated the release of proinflammatory (TNF-α, IL-6) and anti-inflammatory (IL-10) interleukins in Aβ-induced HMC3 cells. Bars represent the release (pg/mL) of TNF–α (**A**), IL–6 (**B**), and IL–10 (**C**) in the presence of the drugs at the indicated concentrations. Data represent means ± S.E.M. from three independent experiments (*n* = 3), performed in duplicate. Statistical analysis was performed by ordinary one-way ANOVA followed by Dunnett’s multiple comparison test. For each experimental condition a selected specific pattern has been used.

Having assessed the cytoprotective effects of T1AM, we moved on to examine the ability of T1AM to suppress β-amyloid-induced upregulation of proinflammatory cytokines production. Exposure of HMC3 cells to 10 μM Aβ25–35 for 24 h promoted a significant increase in the secretion of common proinflammatory cytokines (TNF-α and IL-6), and no effect on the release of anti-inflammatory cytokine IL-10 (Figure 9A–C). Then, we repeated the experiment, exposing HMC3 cells to pretreatment with 1 and 10 μM T1AM. Compared with the Aβ-treated group, the levels of TNF-α and IL-6 were found to show a significant return to normal levels in the group receiving the combined Aβ25–35 and T1AM treatment (Figure 9A,B). In addition, pretreatment with T1AM significantly increased the secretion of anti-inflammatory cytokine IL-10 (Figure 9C). Taken together, these findings indicated the potential of T1AM to inhibit Aβ-induced microglia activation. Since TAAR1 has previously been found to mediate the beneficial effects of T1AM on the inflammatory response of LPS/TNFα-treated HMC3 cells, we subsequently proceeded to examine the role of TAAR1 in mediating the anti-inflammatory effects of T1AM in β-amyloid-induced HMC3 cells. 

We observed that T1AM’s protective effect against Aβ-induced microglia activation was abolished by co administration of TAAR1 selective antagonist EPPTB (1 μM) (Figure 9A–C).

Dysregulation of the transcription factor NF-κB has been widely associated with AD, leading to glial cells activation and neuroinflammation [51]. More importantly, the inactivation of microglial NF-κB has been shown to restore cognitive deficits and help to reestablish a homeostatic phenotype in microglia [52]. 

**Figure 10:**
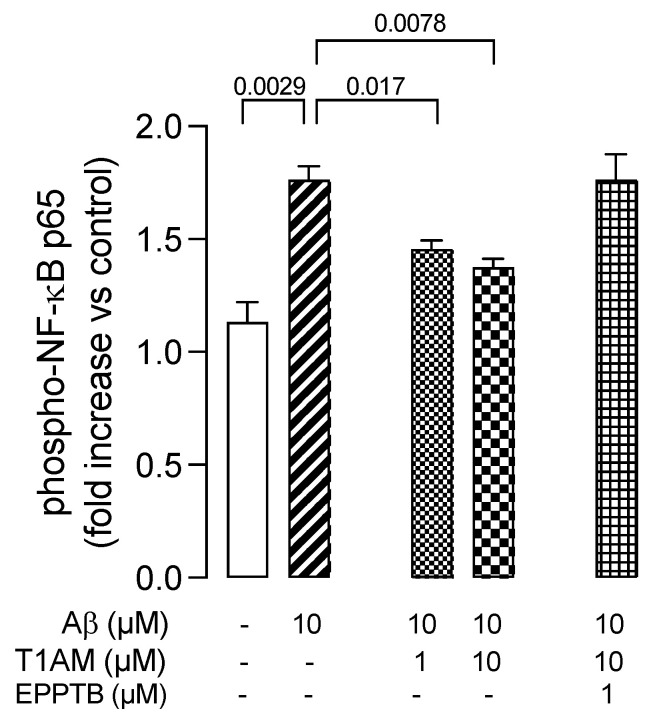
TAAR1 activation modulated the phosphorylation of NF–kB (NF–kB p65) in Aβ–induced HMC3 cells. Data represent means ± S.E.M. from three independent experiments (*n* = 3), performed in duplicate. Statistical analysis was performed by ordinary one-way ANOVA followed by Dunnett’s multiple comparison test. For each experimental condition a selected specific pattern has been used.

Therefore, we decided to evaluate the involvement of the NF-κB signaling pathway in the beneficial effects observed with T1AM in β-amyloid-induced HMC3 cells. Therefore, we repeated key Aβ25–35 and T1AM cotreatment experiments and evaluated NF-κB activity. The cells were then collected, and phospho-NF-κB P65 (p-P65) was detected in the cell lysates (Figure 10). The results showed that Aβ25–35 treatment increased the phosphorylation level of P65 to activate the NF-κB pathway, and T1AM suppressed this response (Figure 10). Notably, in the presence of TAAR1 selective antagonist EPPTB (1 μM), the effect of T1AM on NF-κB activation was completely abolished (Figure 10).

## 3. Discussion 

Increasing evidence supports the concept that T1AM/TAAR1 signaling is part of an endogenous system that can be modulated to prevent neurodegeneration [16,18,32,41,53,54]. AD is the most frequent neurodegenerative disorder in the elderly, usually characterized by memory deficits and cognitive decline. Cognitive function is an important determinant of quality of life, especially in the elderly. The progressive increase in circulating proinflammatory cytokines in inflammaging has been associated with age-related cognitive decline [55] as well as enhanced neuroinflammation, neurodegeneration, and brain release of cytokines by the microglia, which act as resident phagocytic inflammatory cells in the brain [56]. Microglial cells are vital in recruiting these inflammatory mediators, and dysregulated microglial activation represents a neuropathological hallmark in AD and many other NDDs [57]. Microglial TAAR1 signaling has not yet been fully defined, but considerable overlap exists between the immune activated state and TAAR1 expression [29,58,59]. Pathological changes in endogenous TAAR1 agonists, as observed in psychiatric disorders, could underlie alterations in microglial functions [23,60]. Therefore, ascertaining the ability of T1AM, and endogenous agonist for TAAR1 will lead to a better understanding of the role of TAAR1 as a modulator of microglia dysregulation, further expanding the potential of drugs that interact with TAAR1 in the management of AD.

We showed here that T1AM was able to reduce LPS/TNFα-induced IL-6 in HMC3 human microglial cells, while increasing the production of anti-inflammatory interleukin IL-10 without affecting the cells’ viability. In addition, in LPS/TNFα-induced HMC3 cells, treatment with T1AM was observed to decrease the expression of inflammatory response-related genes, including the monocyte chemoattract protein-1 (MCP1), the macrophage inflammatory protein-1 (MIP1), and the transcription factor NF-kB.

Regarding the mechanism of action, T1AM does not bind the nuclear thyroid hormone receptors (TRs) but it stimulates with nanomolar affinity TAAR1, a G protein-coupled receptor that recently emerged to have a role in immunomodulation [53] and neuroinflammation [32]. Nevertheless, there is currently not much information regarding TAAR1 expression in microglia [29]. In the present study, by performing quantitative real-time PCR analysis (qPCR), we demonstrated that TAAR1 is expressed in the human microglial HMC3 cell line, and we found that after LPS/TNFα stimulation, no changing levels of TAAR1 expression were observed in HMC3 cells.

Using a pharmacological approach, we demonstrated that inhibition of TAAR1 was sufficient to prevent T1AM’s anti-inflammatory effect on HMC3 cells, and, conversely, the stimulation of TAAR1 in the absence of T1AM was able to attenuate the LPS/TNFα-induced release of proinflammatory cytokines, as observed in T1AM-treated cells.

In this investigation, the pharmacokinetics results showed that T1AM was rapidly taken up by HMC3 cells and catabolized by oxidative deamination to TA1, which was largely released from cells. 

Even though TA1 is not a ligand of TAAR1 [61], previous works have reported that TA1 may contribute, at least in part, to some effects elicited after the administration of exogenous T1AM [41,43,44,45,62]. Therefore, we checked whether TA1 administration could also decrease the inflammatory phenotype of LPS/TNFα-stimulated HMC3 cells, but obtained negative results, confirming the specific anti-inflammatory effect of T1AM.

A great number of studies have documented the ability of Aβ fibrils to directly stimulate microglia in vitro to assume a neurotoxic phenotype characterized by secretion of a plethora of proinflammatory molecules, ultimately leading to neuron loss [63,64]. We found that aggregated Aβ25–35 peptide (10 μM, 24 h) significantly decreased viability of HMC3 microglial cells. This was concentration-dependently attenuated by T1AM (0.1–10 μM). Pretreatment with T1AM also suppressed the release of TNFα and proinflammatory IL-6 while increasing anti-inflammatory IL-10 levels. These effects were almost completely abolished by treatment with the TAAR1 antagonist EPPTB. In addition, Aβ induced activation of microglial NF-κB by phosphorylation. Application of T1AM attenuated this effect via TAAR1 activation, suggesting that the T1AM/TAAR1 system may protect microglial cells against Aβ-induced cell injury by inhibition of inflammation. 

In summary, we provided, for the first-time, preliminary evidence that the T1AM/TAAR1 signaling pathway has the potential to normalize factors that regulate microglia-mediated neuroinflammation, further increasing the relevance of this system in the pathophysiology of aging-related brain diseases like AD. In animal models, neuroinflammation has been closely tied to impaired function of the hippocampus, associated with a loss of hippocampal pyramidal neurons and entorhinal cells, leading to the disruption of long-term potentiation (LTP) in hippocampal synapses [65,66]. Notably, the T1AM/TAAR1 system has been previously shown to restore LTP in the enthorinal cortex (EC) of mouse models of AD, suggesting that T1AM and TAAR1 are part of an endogenous system that can be modulated to prevent synaptic and behavioral deficits associated with Aβ-related toxicity [41]. The results of the present study may help to elucidate the mechanisms by which the T1AM/TAAR1 system exerts a neuroprotective effect, and the investigation of the relationship with neuroinflammation markers could bring the story one step ahead. These preliminary data are strictly necessary to provide the basis for the ongoing new in vivo study, which we are conducting to clarify the contribution of neuroinflammation to altered hippocampal neuroplasticity, as observed in mouse models of AD. Furthermore, the identification of cytokines specifically associated with cognitive decline may provide novel biomarkers for the development of interventions to slow or reverse AD.

## 4. Materials and Methods

### 4.1. Drugs

T1AM and the TAAR1 antagonist N-(3-ethoxyphenyl)-4-(pyrrolidin-1-yl)-3-trifluoromethyl-benzamide (EPPTB) were purchased from Sigma-Aldrich (Milan, Italy); the TAAR1 agonist (S)-4-[(ethylphenylamino) methyl]-4,5-dihydrooxazol-2-ylamine (RO5166017) was kindly provided by Dr. Gainetdinov. T1AM metabolite 3-iodothyroacetic acid (TA1) was kindly provided by Dr. Scanlan. Aliquots were stored at −20 °C in DMSO as a 200 mM stock solution and diluted to the desired final concentration in culture media.

### 4.2. Analysis of T1AM and TA1

T1AM and its metabolite 3-iodothyroacetic acid (TA1) were assayed in samples by tandem mass spectrometry coupled to liquid chromatography (LC–MS/MS) by using a previously established procedure that allows the simultaneous detection of T1AM and TA1 in each sample [20,40]. T1AM and TA1 intracellular concentrations were also assayed by exposing cell lysate samples to the same LC–MS/MS procedure. 

Briefly, aliquots (0.1 mL) from each sample collection were spiked with 10 μL of a suitable mixture of internal standards (deuterated T1AM and TA1). After adding methanol (0.4 mL), the samples were shaken for 10 min and centrifuged at 22,780× *g* for 10 min. The supernatant was dried under a gentle stream of nitrogen, reconstituted with water/methanol mixture (70/30 by volume), and injected into the LC–MS/MS system. The latter included an Agilent 1290 UHPLC system (Santa Clara, CA, USA) coupled to an AB-Sciex API 4000 triple quadrupole mass spectrometer (Concord, ON, Canada).

### 4.3. Cell Cultures and Reagents

The human microglial clone 3 cell line (HMC3) (ATCC^®^ CRL-3304™, Manassas, VA, USA) was cultured in high-glucose DMEM supplemented with 10% FBS, streptomycin (100 g/mL), and penicillin (100 U/mL) (Sigma-Aldrich, Milan, Italy).

LPS (*Escherichia coli* 0111:B4), TNFα, and Amyloid β-Peptide 25–35 (Aβ25–35) were purchased from Sigma-Aldrich (Milan, Italy).

### 4.4. MTT (Cell Viability Assay)

HMC3 cells were exposed to cell viability assays by using 3-(4,5-dimethylthiazol-2-yl)-2,5-diphenyltetrazolium bromide (MTT, Sigma-Aldrich, Milan, Italy) reagent. Briefly, HMC3 cells were exposed to increasing concentrations of T1AM (0.1–10 µM) and incubated at 37 °C for 24 h. Then, 0.5 mg/mL MTT reagent was added to each well, and the cells were incubated for 3 h at 37 °C. Next, 25 µL of the medium was removed from the wells, and 50 µL of DMSO was added. After incubating for 10 min at 37 °C, absorbance at 540 nm was determined with an automated microplate reader (BIO-TEK, Winooski, VT, USA). The percentage of cell viability was calculated as a percentage of vehicle-treated cells used as control. The same procedure was also followed to detect the cytotoxic effect produced in HMC3 cells after incubation with Aβ25–35 (1 and 10 µM, for 24 or 48 h) in the absence and presence of increasing concentrations of T1AM (0.1, 1, and 10 μM). 

### 4.5. Release of Inflammatory Molecules HMC3 Cells Treated with LPS/TNFα

Proinflammatory IL-6 and anti-inflammatory IL-10 levels were evaluated by specific ELISA assays (RAB0306 (IL-6) and RAB0244 (IL-10), Sigma-Aldrich, Milan, Italy) on collected culture media. Briefly, HMC3 cells were exposed to pretreatment with each of the compounds under investigation (i.e., T1AM, TA1, or RO51660170) for 1 h followed by treatment for 24 h with both LPS (10 μg/mL) and TNFα (50 ng/mL), generally indicated as LPS/TNFα, and used as proinflammatory stimuli. Vehicle-treated cells were used as control. In competition experiments, the TAAR1 antagonist EPPTB (1 μM) was administered 15 min before proceeding with the administration of the selected TAAR1 agonist, namely, T1AM or RO5166017.

### 4.6. Release of Inflammatory Molecules from Aβ25–35-Induced HMC3 Cells

The inflammatory response of HMC3 cells to Aβ25–35 (Sigma-Aldrich, Milan, Italy) exposure (10 µM, 24 h) was evaluated by performing specific ELISA assays. In addition to IL-6 and IL-10 measurements, performed as described above, the levels of tumor necrosis factor TNFα (RAB1089, Sigma-Aldrich, Milan, Italy) and nuclear factor kB (NF-kB) (85-86082-11, ThermoFisher Scientific, Carlsbad, CA, USA) were also evaluated in collected culture media and in cell lysates, respectively, following the corresponding manufacturer’s instructions. Briefly, HMC3 cells were exposed to pretreatment with T1AM (1 or 10 µM) for 1 h followed by Aβ25–35 (10 µM for 24 h), used as proinflammatory stimuli. Vehicle-treated cells were used as control. In competition experiments, the TAAR1 antagonist EPPTB (1 μM) was administered 15 min before proceeding with the administration of T1AM (10 µM).

### 4.7. Gene Expression Analysis

Total RNA was extracted from HMC3 cells using the RNeasy Mini kit (74104, Qiagen, Hilden, Germany) following the manufacturer’s protocol. RNA concentration and purity were determined by Nanodrop-1000 spectrophotometer and Qubit v.1 fluorometer plus Qubit RNA HS Assay Kit (Thermo Fisher Scientific, Wilmington, DE, USA).

Total RNA (1 μg) was retrotranscribed into first-strand cDNA by using by iScriptTM gDNA Clear cDNA Synthesis Kit (Bio-Rad, Milan, Italy) following manual protocol indications. 

Relative quantity of gene transcripts was measured by real-time PCR on samples’ cDNA using an SYBRGreen chemistry and an CFX Connect Real-Time PCR Detection System (Bio-Rad, Milan, Italy). The PCR cycle program consisted of an initial 30 s denaturation at 95 °C followed by 40 cycles of 5 s denaturation at 95 °C and 15 sec annealing/extension at 60 °C. A final melting protocol with ramping from 65 °C to 95 °C with 0.5 °C increments of 5 s was performed for verification of amplicon specificity and primer dimer formation.

Primers were designed with Beacon Designer Software v.8.0 (Premier Biosoft International, Palo Alto, CA, USA) with a junction primer strategy whenever possible. In any case, negative control of retrotranscription was performed to exclude any interference from residual genomic DNA contamination. The primer sequences for real-time PCR are reported in Table 2.

All reactions were performed in triplicate and the amount of mRNA was calculated by the comparative critical threshold (CT) method. To account for possible variations related to cDNA input or the presence of PCR inhibitors, the endogenous reference gene GAPDH was simultaneously quantified for each sample, and data were normalized accordingly.

### 4.8. Statistical Analysis

Statistical analyses were performed using GraphPad Prism version 9.0 for Mac (GraphPad Software, San Diego, CA, USA). Data were subjected to *t*-tests or one-way ANOVA. Significant differences among different treatments were calculated according to Dunnett’s multiple comparisons test. Data are reported as mean ± SEM. Differences at *p* < 0.05 were considered statistically significant.

## Figures and Tables

**Table 1:** LC–MS/MS measurement of T1AM and TA1 levels in HMC3 cells.

Time (min)	Medium		Cell Lysates	
	T1AM (nM)	TA1 (nM)	T1AM (nM)	TA1 (nM)
0	N.D.	N.D.	N.D.	N.D.
5	64.1 ± 2.70	0.31 ± 0.020	26.0 ± 0.28	0.06 ± 0.01
15	57.2 ± 5.40	0.66 ± 0.20	24.9 ± 0.74	0.12 ± 0.02
30	56.7 ± 8.10	1.33 ± 0.15	27.0 ± 0.30	0.14 ± 0.02
60	53.4 ± 3.60	3.81 ± 0.80	24.5 ± 0.81	0.23 ± 0.04

Concentrations of T1AM and TA1 in medium and cell lysates after 0, 5, 15, 30, and 60 min of infusion. Data represent mean ± SEM, *n* = 3 per group, and are expressed as nM. T1AM or TA1 contents were measured in medium and lysate HMC3 cells, which were incubated for 0, 5, 15, 30, and 60 min with T1AM (0.1 µM). N.D., not detectable.

**Table 2:** Primer sequences of target genes.

Reference Sequence (RefSeq) RNA	Gene Symbol	Primer Sequences
NM_002046	GAPDH	(F) 5′-CCCTTCATTGACCTCAACTACATG
		(R) 5′-TGGGATTTCCATTGATGACAAGC
NM_002982.4	MCP1	(F) 5′-GAGAGGCTGAGACTAACC
		(R) 5′-TGATTGCATCTGGCTGAG
NM_002983	MIP1	(F) 5′-ACTTTGAGACGAGCAGCCAGTG
		(R) 5′-TTTCTGGACCCACTCCTCACTG
NM_001404662	NFKB	(F) 5′-CCTTTCTCATCCCATCTTT
		(R) 5′-CCTCAATGTCCTCTTTCTG
NM_138327	TAAR1	(F) 5′-GAGATCTGCTGAGCACTGTTGG
		(R) 5′-CAGCATAGTAGCGGTCAATGGAG

## Data Availability

Data is contained within the article.
